# Beyond neurons and spikes: *cognon*, the hierarchical dynamical unit of thought

**DOI:** 10.1007/s11571-023-09987-3

**Published:** 2023-07-11

**Authors:** Mikhail Rabinovich, Christian Bick, Pablo Varona

**Affiliations:** 1 BioCircuits Institute, University of California, San Diego, USA; 2 Department of Mathematics, Vrije Universiteit, Amsterdam, The Netherlands; 3 Dpto. de Ingeniería Informática, Escuela Politécnica Superior, Universidad Autónoma de Madrid, Madrid, Spain

**Keywords:** Cognitive dynamics, Heteroclinic neural dynamics, Metastable neural states, Brain rhythms, Cognitive binding

## Abstract

From the dynamical point of view, most cognitive phenomena are hierarchical, transient and sequential. Such cognitive spatio-temporal processes can be represented by a set of sequential metastable dynamical states together with their associated transitions: The state is quasi-stationary close to one metastable state before a rapid transition to another state. Hence, we postulate that metastable states are the central players in cognitive information processing. Based on the analogy of quasiparticles as elementary units in physics, we introduce here the quantum of cognitive information dynamics, which we term “cognon”. A cognon, or dynamical unit of thought, is represented by a robust finite chain of metastable neural states. Cognons can be organized at multiple hierarchical levels and coordinate complex cognitive information representations. Since a cognon is an abstract conceptualization, we link this abstraction to brain sequential dynamics that can be measured using common modalities and argue that cognons and brain rhythms form binding spatiotemporal complexes to keep simultaneous dynamical information which relate the ‘what’, ‘where’ and ‘when’.

## Introduction: the cognon concept

Experiments have shown that cognitive activity can be seen as a transient sequential switching across different metastable states, e.g. see the reviews (Tozzi et al. 2017; He 2018; Michel and Koenig 2018; Rabinovich et al. 2020). These states arise on three levels of the brain hierarchy: neuronal, micro-network, and large-scale functional networks. Cognitive dynamics involve many different neural processes and resources including perception, memory, decision making, attention and emotion. In spite of the enormous variety of these processes, which involve multiple brain regions and coordination mechanisms (Tognoli et al. 2021), they demonstrate an amazing universality from the dynamical point of view (Rabinovich et al. 2012b): Within a wide range of measurement modalities and spatiotemporal scales, one observes sequential dynamics between transient metastable states.

Cognitive dynamics can therefore be interpreted as robust sequential switching described by associated metastable sets on different levels of their winnerless competition spatio-temporal activity (see Box 1). The dynamics of these processes can also be described by basic universal models from this viewpoint. In this paper, we introduce the concept of cognon as the unit of thought, which is represented by a robust finite chain of metastable states. Many mathematical models naturally provide the existence of not only metastable states, but also the associated hierarchy of their chains, which serve to describe cognon dynamics. Below we will illustrate this with generalized Lotka–Volterra and Ginsburg–Landau models.

BOX 1. Cognon hierarchy: phase portraits

**Table:** 

	Panel A: Landscape metaphor for a heteroclinic chain consisting of two metastable states located at the simultaneous local minima and maxima of the landscape where the little balls are depicted. A cognon is represented by a robust finite chain of metastable states. A convenient basic model to understand this concept is the Lotka–Volterra model, see Box 4.
	Panel B: Time series of binding dynamics showing the heteroclinic modulation of four modalities. This dynamics naturally emerges in Lotka–Volterra models (Rabinovich et al. 2014).
	Panel C: Cartoon of the phase-portrait of a three-level hierarchy heteroclinic dynamics. As their dynamical robustness is kept in multiple spatio-temporal scales, an arbitrary number of cognons can scale up in information complexity to shape complex cognitive processes.
	Panel E: Schematic phase portrait of a three-modality binding. Each rib of the corridor corresponds to different processing modalities, which are integrated by the heteroclinic interaction (Rabinovich et al. 2010; Afraimovich et al. 2015).
Robust transient cognition can be described as the result of sequential heteroclinic switching determined by a winnerless competitive (WLC) interaction of functional participants (Rabinovich et al. 2008b, 2012b; Afraimovich et al. 2012). WLC is a general dynamical phenomenon that robustly generates sequential switching of prevalence among participants. A simple intuitive example is the game “rock–paper–scissors”.

## Neural dynamics of memory and binding

Episodic memory (EM)—as an example of a cognitive process—contains various details of an event, such as the objects or people involved (“what”), the spatial setting (“where”) and the temporal sequence (“when”) in which the event unfolded. To create an EM representation, the spatiotemporal information must be remembered as a coherently bounded sequence of episodes. For example, the art of music involves balance between surprise and predictability. It is possible to consider music generation as a three dynamic modality process: melody, harmony and rhythm.

Episodic memory is consciously recollected in *spatio-temporal neural activity patterns* related to personally experienced groups of events, i.e., episodes (e.g. see Bergström et al. 2013; Ekstrom and Ranganath 2018). Episodic memory retrieval is a dynamic process that draws upon the sequential ability to reconstruct past experiences from corresponding cues. The neural substrates of these abilities are engrams, which are sets of basic units of memory in the form of mini-networks of neuronal clusters (Kitamura et al. 2017).

Binding is a key dynamical mechanism for the implementation of autobiographic episodic memory (Gilbert et al. 2014; McGatlin et al. 2019); see also Box 2. Binding is the process by which frequently repeated segments of temporal inputs are concatenated into single conceptual units that are easy to process (Gobet et al. 2001). Such processing of information is fundamental to time-series analysis in biological and artificial neural computation systems. The brain efficiently acquires integrated information from various modality streams in an unsupervised manner.

BOX 2. Chain of cognons, binding, and the internal train of thoughts The internal train of thoughts, i.e., the language of thoughts (Fodor 1975), is the result of a cooperation between autobiographical information provided by the default mode network and a frontal–parietal network (Martinon et al. 2019). Episodic and semantic memory are key ingredients for the train and the source of the language of thoughts (Mahr 2020). Episodic memory strategically organizes information in chunks as a function of shared visual, spatial, or temporal characteristics (Gilchrist 2015). In 1956, Miller hypothesized that working memory capacity is not fixed but depends on the strategy, in particular, binding expands the memory capacity (Sala and Gobet 2019). In the language of cognons, the train of thoughts can be considered as a sequence of cognitive informational units. Thus, a dynamical approach to the analyses of the robustness of information integration with other modalities is a natural way to assess these problems. The analysis of the interaction between different modalities of thinking (i.e., episodes from autobiographic memory and self-generation of new thoughts) requires modeling attentional control (Rabinovich et al. 2015). This can be achieved by mathematically describing cognon competition and binding. Such processes are critically important for effective decision making and for increasing the capacity of working memory.

In the global cognitive phase space, sequential memory is represented as trajectories along a chain of metastable states and cognons (at each level of the hierarchy with increasing dimension). In a modeling work (Fonollosa et al. 2015), the authors have shown the learning of such representation of sequences and their robust recall. During learning, the dynamics binds a set of modes to each information-carrying item in the sequence and encodes their relative order. In the recall process, hierarchical WLC guarantees the robustness of the sequence order when the sequence is not too long. Reduced top-down control of cognon interactions could also benefit creative processes. Recently, frontal oscillatory activity in the theta range (4–7 Hz) has been associated with a broad range of top-down control processes. For instance, in the memory domain mid-frontal theta rhythms have been proposed as an inhibitory mechanism modulating the competition between representations during memory retrieval (Hsieh and Ranganath 2014). In Hahn et al. (2019), authors suggest that slow (e.g., alpha-band and theta-band) oscillations and fast (gamma-band) oscillations can serve as an important control mechanism that allows or prevents signals to be routed between specific networks. Slow oscillations can modulate the time required to establish network resonance or entrainment and, thus, regulate the communication between large scale cognitive networks (Siebenhühner et al. 2016).

The functionality of episodic memory, as part of a content and temporal hierarchy in human memory, is not stable across situations; it varies dynamically with the demands of the retrieval context (Fingelkurts et al. 2003a). The hippocampus plays a fundamental role in episodic memory formation. The hippocampal-cortical connections reconfigure during episodic retrieval, and these dynamic interactions might flexibly support the multimodal nature of remembered events (Cooper and Ritchey 2019). One of the important functions of subcortical-cortical connectivity is to continuously update retrieved long-term memory during reconsolidation, creating a constantly evolving emergent engram (Devan et al. 2018).

To remember an event, it is necessary to integrate different modalities (multisensory events) into a coherent representation during the initial encoding, which can be implemented through a heteroclinic binding process (Rabinovich et al. 2010). Episodes unfold across time and contain multiple events. The binding of excited multisensory elements is most likely mediated by fast-acting long-term potentiation (LTP), which relies on the precise timing of neural activity (Markram et al. 1997). In Berens and Horner (2017), authors showed that the hippocampus controls such timing and human episodic memory formation depends on phase synchrony between different sensory networks in the theta frequency band. Synchronization also supports the neural process of constantly encoding new information and integrating it into the existing episodic memory representations (Köster et al. 2019), see also Hanslmayr et al. (2016) and Box 3.

BOX 3. Metastable oscillatory states and transient synchronization in the brain Metastable states are the basic dynamical elements that participate in the formation of transient cognitive activities (Rabinovich et al. 2012a; Deco et al. 2017; Roberts et al. 2019; Alderson et al. 2020). Brain waves, i.e., rhythms, including metastable EEG microstates that are observed also in resting state networks, participate in the generation of different cognitive functions like episodic memory, visuospatial attention, decision making and learning (Kelso 1995; Bressler and Kelso 2001; Fingelkurts and Fingelkurts 2004; Michel and Koenig 2018). The mechanisms for the dynamical interaction between different oscillatory metastable brain modes depend on their spatiotemporal scales (Moyal and Edelman 2019). Here we emphasize as an example the role of the alpha rhythm, which dominates in EEG. By intracranial recordings authors in Halgren et al. (2019) showed that the alpha wave propagates from higher-order to lower-order areas. These results suggest how alpha can coordinate cognitive dynamics throughout the brain; see also Zhang et al. (2018). Episodic memories and predictive coding are related to the processing of distinct time scales and multimodality information and its binding into a coherent, memorable representation (Alamia and VanRullen 2019). These processes are possibly supported by neocortical alpha/beta desynchronization and hippocampal theta/gamma synchronization helping the creation of episodic memories. Some authors have hypothesized that this coupling reflects the flow of information from the neocortex to the hippocampus during memory formation, and the hippocampal pattern completion inducing information reinstatement in the neocortex during memory retrieval (Alamia and VanRullen 2019). Transient synchronization and entrainment are the main dynamical phenomena responsible for the realization of cognitive functions. In particular, alpha/beta desynchronization and hippocampal theta/gamma synchronization represent two separable processes in episodic memory—remembering and recall (Griffiths et al. 2019). Entrainment usually entails phase alignment of brain oscillations (phase entrainment), but can also be present as the alignment of generated events or bursts. In this scenario, the oscillating bursts may result from cross-frequency interactions through which the phase of lower frequency oscillations modulates the amplitude of higher frequency oscillations—phase-amplitude coupling of neuronal oscillations (Bergmann and Born 2018; Fagerholm et al. 2020).

In the context of connectivity and cross-frequency coupling studies, we can hypothesize that brain rhythms influence cognon dynamics by controlling the elements involved in the inhibition. This mechanism can regulate the encoding and retrieval of a series of events inside the cognon, and also in the cognon sequence, i.e., the episodic memory (Zarghami and Friston 2020). A similar mechanism is responsible for the robustness of the multimodality cognitive processes, and it is named heteroclinic binding (Varona and Rabinovich 2016), which is a hierarchical process in transient activity that integrates different sensory or cognitive modalities. Its mathematical image in the cognitive space can take, for example, the form of a heteroclinic cylinder with one or several ribs corresponding to the different modalities.

## Cognon and brain rhythms

Cognitive brain activities can be represented by the dynamics of two qualitatively different components: (1) the specific activity of cognitive networks, and (2) the overall dynamics of continuous oscillatory fields, i.e., brain rhythms. These components process cognitive information in different ways and are often considered independently. However, their mutual interaction through synchronization and desynchronization creates a universal processor with a unique ability to operate with different forms of cognitive mechanisms (see Box 4). The study of such interactions can also help to the assessment of mental disorders (Rabinovich and Varona 2017; Fingelkurts and Fingelkurts 2019). Their associated metrics and parameters that control their relationship can be convenient biomarkers and contribute to novel rehabilitation protocols (Latorre et al. 2019).

To our best knowledge, a consistent mathematical model based on the mutual interaction of both these components does not exist yet. We suggest here for the first time a model of “cognon-field” information dynamics, which we describe by linking a formulation of heteroclinic dynamics—saddle invariant sets in the cognitive phase space connected by heteroclinic orbits—for competitive cognitive interactions and complex Ginzburg–Landau fields (see Box 4).

To build a nonlinear theory of, for example, autobiographic memory dynamics, it is necessary to create a universal scale-free model—a canonical model—of cognitive dynamical processes. We suppose that the canonical model has to satisfy the following conditions: (a) The equations have to be written for variables that can represent the evolution of brain oscillatory clusters in their temporal coherence and have to have solutions that correspond to metastable patterns in the brain; (b) the model is based on winnerless competitive dynamics—a nonlinear process of interaction of many agents that guarantees the sequential switching between metastable states and the robustness of transients, (c) the model is an open dissipative system where inhibition is balanced by excitation, (d) the model’s dynamics have to be sensitive to the incoming information, and (e) be able to describe closed heteroclinic chain dynamics**.** The mathematical image of the cognon is a Stable Heteroclinic channel (SHC) that consists of a sequence of metastable states (see Box 1).

The reduction of high-dimensional brain data to a low-dimensional cognitive space can be motivated by empirical observation. There are a number of experiments that have illustrated the low-dimensionality of cognitive dynamics when it is governed by sensory stimuli (Shine et al. 2019). Formally, this means that large amounts of data can be represented by the dynamics of a reduced number of spatiotemporal patterns—or modes—e.g., using spatiotemporal decomposition techniques, see Banerjee et al. (2012), Pinotsis et al. (2014) and Glomb et al. (2017).

In terms of EM, the formation and retrieval of event memories are implemented by collaborative dynamics between the neocortex and the hippocampus, as can be observed by the analysis of neocortical alpha/beta frequency desynchronization and hippocampal theta/gamma frequency synchronization (Griffiths et al. 2019). In this task, the neocortex processes event-related information and the hippocampus binds this information. Such brain rhythm analyses indicate that a bidirectional information exchange between the neocortex and the hippocampus is fundamental for the formation and retrieval of episodic memories.

The learning dynamics—forcing of sequential events by the environment—activates the chain of engrams in time. On the retrieval stage, this chain of engrams replays robustly through the sequential competitions between individual engrams in time (Rashid et al. 2016; Rao-Ruiz et al. 2019; Takamiya et al. 2020) and thereby reconstructs the original sequence of events.

As an example, let us consider creative cognition or goal directed self-generated creative thought (Beaty et al. 2016). First, it necessary to consider the dynamic interaction of large-scale brain networks and their constituting processes that participate in creative tasks. These dynamical processes are: (1) creativity, idea generation, and elaboration, (2) sequential working memory, (3) attention, and cognitive correlation and control. Creative idea generation is based on interactions including frontal-central as well as frontal–temporal networks (Rominger et al. 2020). Variables, i.e., amplitudes and phases that describe the different modes of this network form the cognitive phase space where there exists a heteroclinic structure based on metastable states (Rabinovich et al. 2008b), the mathematical image of cognons. The complexity of the functional connection matrix (see Eq. (2) in Box 4)—depends on personality and the stage of the creativity process (Fink and Benedek 2014). At the same time, the creativity dynamics, including its constituting functional components, like working memory (de Vries et al. 2020), are controlled by brain rhythms in the alpha frequency range. Alpha power helps estimating the creativity level of ideas, and is responsible for the functional correlation of large scale brain activity (Benedek and Fink 2019). Alpha oscillations also play an important role in the organization of divergent thinking and the performance on the alternative uses task (Agnoli et al. 2020), and serial order effect (Kraus et al. 2019). In our model, the power of brain oscillations can control the elements of the connection topology (matrix *ς*_*ij*_ in (2)), which determine the existence of cognons. At the same time, the cognon influences the generation of alpha oscillations making the basic model with such feedback self-consistent.

BOX 4. Cognon: simplest mathematical models Complex patterns of cognitive activity as measured in electrophysiological and imaging experiments can be described in terms of sequential metastable states using the concept of cognon as the dynamical unit of cognitive information. This description allows the characterization of highly coordinated cooperative dynamics in neuronal media and global discrete networks. To implement the dynamics illustrated in Box 1, a sequence of cognons can be mathematically represented in a model as a vector *C*_*j*_ with *M* ordered sequence components (*j* = 1,2,…,*M*) in the form:

1 $$C_{j} (\vec{r},t,\tau ) = \left( {a_{j} (t)e^{{i(\Omega_{j} t + \varphi_{j} )}} + a_{j}^{*} (t)e^{{ - i(\Omega_{j} t + \varphi_{j} )}} } \right)P_{{}}^{j} (\vec{r},\tau )$$

where *a*_*j*_ (*t*) is the level of excitation of the *j*-th component, ${\Omega }_{j}$ is the frequency of synchronized neural clusters-modes on the *j*-th component, ${\varphi }_{j}$ is its phase, $P_{{}}^{j} (\vec{r},\tau )$ represents the spatial structure of the cognon *C*_*j*_, $\vec{r}$ is the space coordinates, and $\tau$ is the characteristic time of the dynamics.

**Complex generalized Lotka–Volterra equations**

As argued above, cognitive dynamics can be described as a kinetic process in the form of a sequence of competitive cognons—finite heteroclinic chains. A convenient model for the description of such type of kinetic process is a complex Lotka–Volterra equation (Varona and Rabinovich 2016). In this model, the joint evolution of amplitude and phase in (1) can be written as

2 $$\tau_{j} \frac{{da_{j}^{{}} }}{dt} = - a_{j}^{{}} \cdot \left[ {\nu - \gamma \left( S \right)\left| {a_{j} } \right|^{2} + \sum\limits_{i = 1}^{M} {\varsigma_{ji}^{{}} \left( S \right)} \;a_{i} a_{i}^{ * } } \right]$$

where *τ*_*j*_ is the time scale of *a*_*j*_, $\nu$ and *γ*(*S*) represent the level of inhibition and excitation (which depends on the stimulus *S*), respectively, and *ς*_*ij*_(*S*) is an inhibitory connection matrix that guarantees winnerless competition dynamics (Rabinovich et al. 2008a). Model (2) describes a sequence of metastable states as a typical characteristic of transient brain activity, which can be identified with several techniques such as EEG and fMRI (Roberts et al. 2019).

**Complex Ginzburg–Landau equations**

The complex Ginzburg–Landau equation (CGLE) (Aranson and Kramer 2002) describes metastable dynamics of localized elements of complex mean fields that can be related to brain rhythms. If we suppose that $u(\vec{r},\tau_{j} )$ is the complex spatiotemporal amplitude of brain oscillations, their dynamics can be described as:

3 $$\frac{\partial u}{{\partial t}} = \left( {Q - (1 + i\beta )\left| u \right|^{2} } \right)u + (1 + i\alpha )\Delta u$$

Here $\Delta u = \frac{{\partial^{2} u}}{{\partial z_{1}^{2} }} + \frac{{\partial^{2} u}}{{\partial z_{2}^{2} }}$, *z*_1,2_ are space coordinates, *Q, α* and *β* are parameters that characterize subcriticality, dispersion and nonlinearity, respectively. Because a collection of brain waves is typically characterized by several different bifurcation frequencies, it is necessary to generalize the model from its classical formulation.

**Coupled cognon dynamics**

GGLVE and CGLE models can be combined for a joint description of cognon heteroclinic dynamics and brain rhythms interaction as a finite perturbation for each of them which does not destroy their basic architecture according to

4 $$\tau_{j} \frac{{da_{j}^{{}} }}{dt} = {\text{r.h.s.}}_{(2)} + \chi u$$

5 $$\frac{{\partial u_{j} }}{\partial t} = {\text{r.h.s.}}_{(3)} + \sigma a(t)$$

where r.h.s._(x)_* is the right-hand side of equation* (x) and *χ* and *σ* are coupling parameters between different components of the (2) CGLVE and (3) CGLE models. For simplicity, we consider here a linear coupling. This joint model is able to represent cognon dynamics and their interaction with brain rhythms to describe and predict robust sequential information processes involved in cognitive functions such as autobiographic memory, attention, decision-making, emotion and behavior. In multimodality cognitive processes, including creative or perceptual tasks, cognons form binding spatiotemporal complexes that keep simultaneous dynamical information that relate the what, the where and the when.

## Conclusions

The concept of a cognon captures the sequential nature of essential cognitive processes, which can also be seen within the framework of a generative model linking continuous and discrete time descriptions of neural activity and its associated behavior (Rabinovich and Varona 2018; Parr et al. 2023). The hierarchical multilevel timing architecture of basic cognon models is a convenient way for analyzing sequential binding phenomena of cognitive dynamics. It can be a sequence of events in the episode or a group of symbols (words) forming a thought. In contrast, with the chunking process of one modality, the process of binding of different perception features—or modalities—requires temporal coordination of parallel transient modalities (Fingelkurts et al. 2003b). For such description, the number of different layers in the model has to coincide with the number of modalities.

The multilayer dynamics of (4) and (5) is also adequate to model decision-making (DM) processes in the case when DM is viewed as a choice between different binding modalities modulated by the environment. This binding occurs in many decision-making tasks related to behavior.

Different physiological signals, as recorded by in vitro, in vivo electrophysiological experiments, fMRI and EEG, capture the same brain activity from different time and spatial scales, and their dynamical interaction (Van De Ville et al. 2010; Bassett and Sporns 2017; Avena-Koenigsberger et al. 2017). Interlaced robust sequential informational processes are observed in subcellular, cellular, small network and large network interactions, including systems interaction. A basic dynamical model has to describe the simultaneous temporal evolution of these signals and their mutual interaction depending on the specific cognitive goal and environment condition. Building sequences of sequences with such dynamical objects naturally gives rise to coordinated hierarchical neural phenomena at multiple description levels, from microscopic to macroscopic information flows, from sensory encoding to cognitive decision making.

The cognon approach described by the above discussed model consists of two groups of equations, i.e., equations for continuous spatio-temporal fields, and equations of cognon dynamics. They interact by mutual modulation of the control parameters including mutual excitation and inhibition. Of course, hypotheses about the robustness and reproducibility of solutions of (4) have to be proved in the future. However, available experimental data about the existence and reproducibility of metastable informational patterns in active brains (Tognoli and Kelso 2014; He 2018; Roberts et al. 2019) support this hypothesis and can guide new experiments, including those related to pathological states (Rabinovich and Varona 2017). The concept of cognon, as the basic unit of cognitive information can help to bridge the gap between theoretical formalisms of cognitive dynamics, physiological measurements and information programming of behavior. Additional impact of this concept can include the realm of artificial intelligence and, in particular, quantum inspired artificial agents (Huber-Liebl et al. 2022).
